# The role of androgens in pressure overload myocardial hypertrophy

**DOI:** 10.3389/fendo.2023.1112892

**Published:** 2023-02-01

**Authors:** Marie Schafstedde, Sarah Nordmeyer

**Affiliations:** ^1^ Department of Congenital Heart Disease – Pediatric Cardiology, Deutsches Herzzentrum der Charité – Medical Heart Center of Charité and German Heart Institute Berlin, Berlin, Germany; ^2^ Institute of Computer-Assisted Cardiovascular Medicine, Charité – Universitätsmedizin Berlin, Berlin, Germany; ^3^ Partner Site Berlin, German Center for Cardiovascular Research (DZHK), Berlin, Germany; ^4^ Berlin Institute of Health at Charité – Universitätsmedizin Berlin, Berlin, Germany

**Keywords:** testosterone, dihydrotestosetrone, hypertrophic, pressure overload, aortic valve stenosis

## Abstract

Pressure overload hypertrophy of the left ventricle is a common result of many cardiovascular diseases. Androgens show anabolic effects in skeletal muscles, but also in myocardial hypertrophy. We carefully reviewed literature regarding possible effects of androgens on specific left ventricular hypertrophy in pressure overload conditions excluding volume overload conditions or generel sex differences.

## Introduction

Pressure overload hypertrophy (POH) of the left ventricle is a common result of diseases like arterial hypertension, aortic valve stenosis, hypertrophic obstructive cardiomyopathy or aortic coarctation and is associated with increased morbidity and mortality (1–4). Thus, many patients of any age and sex are affected and interventions and medical treatment aim to reduce or prevent POH. Common believe that cardiac hypertrophy is a compensatory mechanism still exists. As a short-term response it minimizes wall stress and reduces oxygen consumption, but when persisting for a long time, maladaptive remodeling with proarrhythmic properties and ultimately heart failure occurs. There is increasing literature arguing that hypertrophic response is not specifically needed. In line with these findings Schiattarella et al. suggest that inhibition of ventricular hypertrophy might be a good therapeutic strategy in the treatment of POH (5, 6). Testosterone and its most active metabol-ite Dihydrotestosterone (DHT) are well known for their anabolic effects in skeletal muscles (7–10), however, much less is known about specific effects of both androgens onto the human myocardium, especially in pressure overload conditions. However, in the literature, there is increasing evidence that androgens also contribute to the development and progression of myocardial hypertrophy (11–14) and that reduction of serum androgen levels reduce or prevent myocardial hypertrophy (15–18). This review focusses on the specific question of the possible role of Testosterone and/or DHT in cardiac pressure induced hypertrophy and discusses the topic of anti-androgenic medication as a possible treatment option to prevent or reduce POH and thus improve patient outcome. This short review does not aim to highlight all sex differences in cardiac diseases or other kinds of hypertrophy like volume induced hypertrophy, for example.

## Androgens and cardiac hypertrophy

Testosterone in the human body is mainly produced by the gonads (by the Leydig cells in testes in men and by the ovaries in women) and in smaller amounts by the adrenal glands in both sexes. Conversion to DHT takes place *via* the enzyme 5 alpha reductase (19). Both hormones act *via* androgen receptors (AR), but DHT shows twice the affinity for the AR and a ten-fold more potent effect on signalling pathways when compared to Testosterone (20). Both hormones are present in pre- and postmenopausal women and men (13). In humans and animals, reduction of DHT levels can be achieved by blocking the enzyme 5 alpha reductase (e.g. by Finasteride), for example (15, 16). Total Testosterone and, thus, also DHT can be reduced by blocking central molecules of the endocrine system, namely luteinizing hormone (LH) and Follicle-stimulating hormone (FSH), through Gonadotropin-releasing hormone (GnRH) agonists and/or antagonists (21). A third mechanism to reduce androgen actions is the use of androgen receptor antagonists like used in patients with prostate cancer (22).

### Experimental evidence

In cells, it was shown, that Testosterone and also DHT mediate their effects *via* androgen receptors (AR) and induce myocardial hypertrophy *via* two different ways: 1) in a DNA binding-dependent manner (genomic pathway) where androgens bind to the AR, translocate into the nucleus and act like a transcription factor or 2) in a non-DNA binding-dependent manner (non-genomical pathway) where androgens bind to the AR and activate rapid 2nd messenger signalling cascades, for example (23–27). In neonatal rat cardiac myocytes, for example, Testosterone stimulation lead to GSK-3β inhibition and activation of calcineurin and NFAT, thus, resulting in cardiomyocyte hypertrophy (28, 29). On the other hand, Testosterone stimulation in neonatal and adult cultured rat cardiomyocytes in another study has also been shown to induce a rapid intracellular calcium increase by a non-genomical pathway, which influenced contractility (30).

Several animal models exist, that highlight the AR-mediated influence of Testosterone and DHT on cardiac hypertrophy (14, 31–34). In mice with angiotensin II induced hypertrophy, knockout of AR leads to a significant reduction in cardiac hypertrophy and fibrosis (35), and in a rat model of myocardial infarction, low levels of DHT were described to be protective against cardiac hypertrophy (36). In mice undergoing transverse aortic constriction (TAC) in order to induce left ventricular pressure overload hypertrophy, the reduction of DHT levels by finasteride treatment led to significant reduction in hypertrophy (16). Comparably, anti-androgenic treatment with an AR antagonist Flutamide lead to reduction in cardiac hypertrophy in a rat model of hypertension (18). Additionally, it was shown that treatment with finasteride improved cardiac function, attenuated remodeling and reverted pathologic gene-expression after myocardial infarction in mice (15).

### Clinical evidence

In human myocardium, androgen receptor genes are expressed in female and male cardiac myocytes and Testosterone and also DHT produce an hypertrophic response by acting directly on cardiac muscle cells in non-pressure overload condition (11). There are multiple studies in other non-pressure overload conditions in humans describing a pro-hypertrophic mechanism of androgens. In a large prospective study of 2810 men and postmenopausal women, higher serum levels of Testosterone were associated with a greater increase in left ventricular muscle mass over the course of 9 years in otherwise healthy individuals all with physiological levels of Testosterone (13). Among men with type 1 diabetes and physiological levels of Testosterone there was an association of higher (but still normal) serum levels of Testosterone and higher left ventricular muscle mass (37). Furthermore, women with polycystic ovary syndrome, who suffer from hyperandrogenism, are known to have an increased risk for left ventricular hypertrophy (38). If Testosterone or DHT levels might be responsible for that has not been studied. Additional evidence exists in human athletes, in whom the use of anabolic steroids (as synthetic derivatives of the male sex hormones Testosterone), was also associated with cardiac hypertrophy (39).

## Androgens in human pressure overload hypertrophy and anti-androgen therapy as a possible treatment option

In patients with pressure overload hypertrophy caused by severe aortic valve stenosis for instance, cardiac hypertrophy is associated with increased morbidity and mortality (1–4) and treatment aims to reduce the hypertrophic response to the pro-hypertrophic hemodynamic stimuli. In a recent publication, we have described an association of higher serum DHT levels and increased left ventricular muscle mass in female and male patients with POH due to severe aortic valve stenosis (all patients showed physiological DHT levels) (40). Furthermore, serum DHT levels were also associated with higher left ventricular myocardial protein expression levels of moesin, which is part of the ezrin/radixin/moesin (ERM) complex and activates the cardiac sarcolemmal Na+/H+ exchanger. Na+/H+ exchanger, in turn, is described to be associated with increased left ventricular hypertrophy in animal studies (41–43).

Acknowledging that Testosterone and DHT are associated with cardiac hypertrophy, it seems tempting to think about the use of anti-androgenic therapy to prevent cardiac remodeling in patients with AS to improve outcome in these patients.

In our study cohort, a very small number of four male patients happened to be on anti-androgenic therapy due to concomitant prostate disease (two patients were treated with GnRH analogues showing low levels of Testosterone and DHT and two patients with finasteride treatment, where one patient showed normal DHT levels and one reduced DHT levels compared to the other patients). Next to low serum levels of DHT these patients showed low degree of left ventricular muscle mass despite severe aortic valve stenosis and pressure overload (40). Our findings certainly do not prove any causal relationship, however, they suggest an association between higher serum levels of DHT, increased moesin expression levels and higher degree of cardiac hypertrophy in patients with severe AS. In future studies, mechanistic effects of DHT on cardiac hypertrophy in human patients with POH are needed. Another retrospective, observational study in patients with heart failure has described an association between anti-androgenic therapy with finasteride (in patients with heart failure and prostate disease) and attenuated cardiac hypertrophy (17).

Data from animal studies and the few existing human data suggest that anti-androgenic therapy has the potential to improve cardiac function and remodelling in heart failure and to reduce or prevent cardiac hypertrophy in pressure overload conditions. Next to the detrimental pro-hypertrophic cardiac effects, Testosterone replacement therapy has been associated with prostate cancer, polycythemia and obstructive sleep apnea and many long-term effects are still unknown (44, 45). Thus, anti-androgenic therapy might also have additional positive side effects next to anti-hypertrophic action.

On the other hand, excessive reduction of serum androgen levels should be prohibited, especially in male patients. Very low levels of Testosterone in elderly men with Testosterone deficiency have been associated with increased cardiovascular risk and mortality and positive cardiovascular effects have been described for Testosterone replacement therapy in these patients (46–49). Testosterone replacement therapy was shown to alleviate myocardial ischemia in elderly men with coronary artery disease and Testosterone deficiency (50, 51) and to increase exercise capacity in male patients with heart failure with and without Testosterone deficiency (47, 52, 53), or improve serum glucose levels and hemoglobin A1c values in male hypogonadal patients with diabetes (54, 55). Although these positive effects of Testosterone replacement therapy only aim to achieve normal ranges of serum Testosterone levels and are most likely independent of the hypertrophic effect and rely more on vascular and metabolic effects, they should, however, be considered when thinking of possible anti-androgenic therapy in patients with POH. At the same time we want to mention, that studies about possible positive or negative cardiovascular effects of serum androgen levels rarely exist in women and female and male children, which should be considered in future clinical studies.

The study situation currently remains controversial and future studies are required to determine, whether and if yes what kind of anti-androgenic therapy might be a treatment option at least for time-restricted therapy in patients with severe AS waiting for surgical or interventional relieve of pressure overload.

## Conclusion

Testosterone and/or its active metabolite DHT increase the hypertrophic response of left ventricular myocardium to pressure overload in animal models and associations were seen in human subjects. Anti-androgenic treatment to lower Testosterone and/or DHT levels should be discussed as a possible treatment option to reduce cardiac hypertrophy; however, possible negative effects of low serum levels of Testosterone onto vascular and metabolic health have to be taken into consideration. Future studies should focus on cardiac-specific anti-androgenic therapy in order to prevent myocardial hypertrophy in pressure overload conditions without negatively affecting general patient health in women and men.

## Author contributions

All authors listed have made a substantial, direct, and intellectual contribution to the work, and approved it for publication.
